# A Mental Health Chatbot with Cognitive Skills for Personalised Behavioural Activation and Remote Health Monitoring

**DOI:** 10.3390/s22103653

**Published:** 2022-05-11

**Authors:** Prabod Rathnayaka, Nishan Mills, Donna Burnett, Daswin De Silva, Damminda Alahakoon, Richard Gray

**Affiliations:** Centre for Data Analytics and Cognition, La Trobe University, Bundoora, VIC 3086, Australia; p.rathnayaka@latrobe.edu.au (P.R.); n.mills@latrobe.edu.au (N.M.); d.burnett@latrobe.edu.au (D.B.); d.alahakoon@latrobe.edu.au (D.A.); r.gray@latrobe.edu.au (R.G.)

**Keywords:** chatbot, mental health support, artificial intelligence, behavioural activation, personalised assistance, emotional support, conversational agents, mental health monitoring

## Abstract

Mental health issues are at the forefront of healthcare challenges facing contemporary human society. These issues are most prevalent among working-age people, impacting negatively on the individual, his/her family, workplace, community, and the economy. Conventional mental healthcare services, although highly effective, cannot be scaled up to address the increasing demand from affected individuals, as evidenced in the first two years of the COVID-19 pandemic. Conversational agents, or chatbots, are a recent technological innovation that has been successfully adapted for mental healthcare as a scalable platform of cross-platform smartphone applications that provides first-level support for such individuals. Despite this disposition, mental health chatbots in the extant literature and practice are limited in terms of the therapy provided and the level of personalisation. For instance, most chatbots extend Cognitive Behavioural Therapy (CBT) into predefined conversational pathways that are generic and ineffective in recurrent use. In this paper, we postulate that Behavioural Activation (BA) therapy and Artificial Intelligence (AI) are more effectively materialised in a chatbot setting to provide recurrent emotional support, personalised assistance, and remote mental health monitoring. We present the design and development of our BA-based AI chatbot, followed by its participatory evaluation in a pilot study setting that confirmed its effectiveness in providing support for individuals with mental health issues.

## 1. Introduction

Close to a billion people worldwide have experienced a mental illness, ranging from the most common conditions of anxiety and depression to psychotic and personality disorders [1]. Mental illnesses cause a significant degradation of the affected individual’s quality of life, as well as his/her contribution to society and the economy. The collective negative impact has been measured as a “cost” to the world economy of approximately USD 2.5 trillion per year and projected to rise to USD 6 trillion by 2030 [1]. The urgency of mental healthcare and treatment has been emphasised by the World Health Organization, in its Special Initiative for Mental Health (2019–2023), which aims to “ensure access to quality and affordable care for mental health conditions in 12 priority countries to 100 million more people” [2].

Numerous pharmacologic and non-pharmacologic interventions are prescribed for mental illnesses, including surgical, in-patient, out-patient, medication, support groups, counselling, psycho-social, behavioural, and alternative therapies. The type of intervention depends on the category of illness and its severity. The International Classification of Diseases (ICD) [3] produced by the World Health Organization (WHO) and the Diagnostic and Statistical Manual of Mental Disorders (DSM-5) [4] are formalised taxonomies for categorising mental illnesses using common language and standard criteria. The ICD identifies 11 and the DSM-5 outlines 20 main categories of mental illnesses. Anxiety and depression are the most common mental illnesses that are also most prevalent among working-age people. Psycho-social and behavioural interventions are widely accepted as the non-pharmacologic gold standard for the treatment of anxiety and depression [5,6,7,8,9]. As a problem-focused and action-oriented form of therapy, Cognitive Behavioural Therapy (CBT) is the most researched type, which is superior to other forms of psychotherapy and aligns with the information processing disposition of the human brain [10]. Technological innovations are increasingly used to scale up the delivery of CBT psycho-social and behavioural interventions to the large cohorts of individuals affected. Specifically, CBT has been trialled in computer vs. standard settings [11], as a mobile phone service [12], as an online service on the Internet [13], as a gamified smartphone application [14], and as a smartphone-based conversational agent [15]. Conversational agents (or chatbots) are a recent technological innovation that is available all hours, easily accessed using a smartphone, communicates with any number of individuals, and is not impacted by cognitive biases. Unlike computer-aided tools that require some human expertise, chatbots are online and fully automated, which makes them effective at providing first-level support for mental illnesses. Woebot, a chatbot that delivers CBT, has been shown to be effective across multiple studies in depression [15], anxiety [16], and substance abuse [17]. It has also been reviewed by individual contributors who describe their personal experience with the chatbot service [18,19,20]. This growing body of evidence validates the utility and effectiveness of using chatbots for first-level mental health support.

In contrast to CBT, a new stream of work has positioned Behavioural Activation (BA) as a more effective therapy (across multiple factors) for anxiety [21] and depression [22]. BA actively engages affected individuals with positively reinforcing experiences and decreases avoidance behaviours by detaching the cognitive component. BA is more efficiently understood and implemented by patients and practitioners than CBT [23]. Several studies have confirmed this comparative advantage, specifically when the patient is emotionally supported and the continuous engagement is personalised [24,25,26]. However, low acceptability, stigma towards treatment, organisational climates, and cultures have been cited as challenges to the large-scale implementation of BA [27]. To the best of our knowledge, the role of chatbots for the delivery of BA has not been explored in the extant literature. In this paper, we advance this position by postulating that BA can be effectively materialised using Artificial Intelligence (AI) in an intelligent chatbot setting to provide recurrent emotional support, personalised assistance, and remote mental health monitoring capabilities. The research objectives of this study are (1) a conceptual framework for the materialisation of BA as an intelligent chatbot with continuous personalised engagement and emotional support, (2) the design and development of a prototypical BA-based AI chatbot as a cross-platform smartphone application, and (3) the participatory evaluation of the chatbot in a pilot study setting. The rest of the paper is organised as follows. Section 2 presents related work on chatbots for mental health support. Section 3 and Section 4 articulate the methods of this study, where Section 3 presents the conceptual framework and Section 4 delineates the design and development of the chatbot across personalised conversational features and emotion support features. The participatory evaluation is reported in Section 5, and the paper concludes with Section 6.

## 2. Related Work

Depression and anxiety have been associated with economic inactivity, loneliness, poor physical health, and mortality [28]. Prevalence estimates for depression and anxiety vary considerably between studies depending on how mental health problems are defined and methodologies researchers have adopted. The World Mental Health survey (WMHS), the most comprehensive longitudinal study of mental health globally, estimated that in high-income countries, the lifetime prevalence of depression is around 15% [29]. Half of participants in the WMHS reported at least some symptoms of depression. The World Health Organisation ranks depression as the second leading cause of disability globally [30]. Therefore, it is crucial to explore innovative therapies and interventions to support affected individuals. Practice guidelines generally advocate a stepped approach to depression treatment [31]. These guidelines recommend self-help interventions, such as psycho-education and mindfulness, for individuals experiencing mild symptoms. If symptoms are more severe, then psychological treatments such as CBT are recommended. CBT is based on the theory that how we think affects how we behave and feel. During CBT sessions, the therapist will use a range of strategies and techniques to help challenge an individual’s thinking. There is extensive evidence from systematic reviews and meta-analyses that CBT is a safe and effective treatment for depression [31]. However, CBT is a complex treatment that requires extensive training to be able to competently deliver a high degree of treatment fidelity. Consequently, there is considerable and persistent unmet need for effective psychological depression treatments because of a chronic shortage of trained therapists [32]. To address the unmet needs for treatment, there has been considerable interest in “third-wave psychological interventions”, such as Behavioural Activation (BA). A systematic review and meta-analysis of five randomised controlled trials involving 601 participants has reported equal efficacy between BA and CBT [29].

Conversational agents (chatbots) automate the function of communicative labour, in both oral and written conversations [33]. The proliferation of voice assistant chatbots such as Siri, Alexa, Cortana, and Google [34], as well as the numerous chatbot functions in online retail has familiarised a majority of modern society with the utility, engagement, and operation of a chatbot. For instance, in industrial settings, chatbots are used to provide information, instructions, detect fatigue, and address exceptions [35,36], while in healthcare, chatbots have been used for automated post-treatment communications and support groups, counselling, and healthcare service administrative support [37,38]. However, most chatbots are designed using Frequently Asked Questions (FAQs) for information provision or process specific in executing a well-defined repetitive or sequential series of tasks via conversational inputs. The major challenge for chatbots is in emulating the natural flow of conversation between humans and the exchange of information through this natural dialogue. Natural Language Processing (NLP) techniques and Artificial Intelligence (AI) algorithms have been proposed and leveraged to address this challenge [39,40]. NLP and AI have been used widely in diverse healthcare use cases, as reported by Wang et al. [41].

Recent work on conversational agents in the mental health domain has shown promising developments. The first known chatbot of this kind is ELIZA, which was developed to mimic text-based communication with a psychotherapist [42]. Similarly, conversational agents integrated with Cognitive Behaviour Therapy (CBT) have been successfully used for mental health treatments. Fitzpatrick et al. [15] developed a fully automated conversational agent named “Woebot” to deliver cognitive behaviour therapy to users with symptoms of depression and anxiety. Their study found that the users who used Woebot significantly reduced their symptoms of depression over the study period, as measured by Patient Health Questionnaire 9 (PHQ-9). Woebot has been downloaded over 100,000 times (https://play.google.com/store/apps/details?id=com.woebot, (accessed on 1 January 2022)) on the Google Play store with mostly positive reviews. This application is a frame-based conversational agent, i.e., the conversation flow itself is not static, but the user input fills out slots in a fixed template. A similar app, “Wysa” (https://play.google.com/store/apps/details?id=bot.touchkin, (accessed on 1 January 2022)), was developed by Inkster et al. [43] to determine the effectiveness of delivering positive psychology and mental well-being techniques in a text-based conversational mode. Their study revealed that frequent users of the app had significant improvements compared to those who used the app less frequently. Alqahtani and Orji [44] analysed user reviews from mental health mobile applications and identified design limitations. The key findings of this study were personalisation, affordability, information provision, plain-language explanations of mental health issues, advanced security features such as encryption and de-identification, and the inclusion of emergency contacts. Furthermore, most of the existing apps lack professionally designed user interfaces and user experience (UI and UX) factors.

Melcher et al. [45] conducted a study among college students who use mental health apps and barriers to sustained use. It was found that students appreciated the potential to share information with a clinician and the amount of time saved by using such an application. The privacy and accuracy of recommendations were found to be the concerns of the students. This study outlined that the user interface of the app is one of the main deciding factors of engagement and sustained use. User experience features of the app such as easy navigations, app prompts, notifications. and text input were highlighted as important features. The students preferred a clean and simple user interface. The credibility and reliability of the information provided were also contributing factor for sustained use.

Drawing on the recent discoveries on the efficacy of psychological treatments, recent advances in chatbot, AI, and NLP technologies, and the state-of-the-art in effective chatbot and smartphone app features for mental health support, we propose a conceptual framework for a BA-based AI chatbot in the next section, followed by its design, development, and participatory evaluation in the subsequent sections.

## 3. Conceptual Framework of a BA-Based AI Chatbot

The conceptual framework was formulated using a structured approach consisting of three phases. In the first phase, we critically reviewed the adoption of BA in five studies [46,47,48,49,50] from across the world, as well as the recommendations of the best practices of BA reported by professional and lived-experience expert panels [22,27,51,52]. While this review revealed the challenges of implementing BA using a conversational agent, such as low acceptability, stigma towards treatment, continuous engagement, and recurrent emotional support, it also emphasised the effectiveness of BA, aptly summarised as “Life will inevitably throw obstacles at you, and you will feel down. When you do, stay active. Do not quit. I will help you get active again” by Kanter and Puspitasari [27]. Phase 1 informed the primary constructs of the conceptual framework as the anatomy of engagement, emotion detection, mood transition tracking, activity bank, personalised experiences, positive reinforcements, and third-party intervention. The primary constructs of the conceptual framework are presented in Table 1. The multi-layered relationships between constructs and how they enable the BA-based AI chatbot setting were also devised in this phase.

In Phase 2, the primary constructs were enriched with data-driven insights drawn from online mental health support groups. The materialisation of each construct in online support group environments was extracted and sampled to create content representing each construct and the interlinking between constructs. We leveraged the PRIME framework for this task. PRIME is a novel ensemble of machine learning and deep learning algorithms for classification, clustering, association rules, and NLP techniques that imposes a structure onto unstructured text generated by free-flowing online mental health support group discussions. A single discussion is generally used to discuss various topics by a number of individuals. An online support group will comprise a large number of such discussions. As these discussions continue over time, individuals contribute their decisions, experiences, and opinions. The naturally occurring order of discussions provides a further multitude of granular and aggregate information on patient behaviours and emotion expressions [53,54,55,56]. Empathetic engagement was a further consideration of this phase, where empathetic language conveys a broader spectrum of human caring. This more inclusive definition resolves some boundary overlaps between empathy and related constructs in the literature. In the context of a conversational interface, empathetic engagement means, “making the impression of a credible and trustworthy conversation partner that can hear you out and offer a detached point of view on things” [57]. This data enrichment phase validates the robustness of constructs identified in Phase 1 and enables the interlinking of constructs. Finally, in Phase 3, an expert panel of psychologists, psychosocial support workers, and counsellors were engaged to further validate and synthesise the constructs, contents, and interconnections of this conceptual framework for a BA-based AI chatbot. We conducted two rounds of reviews, followed by model refinement and consolidation.

**Figure 1:**
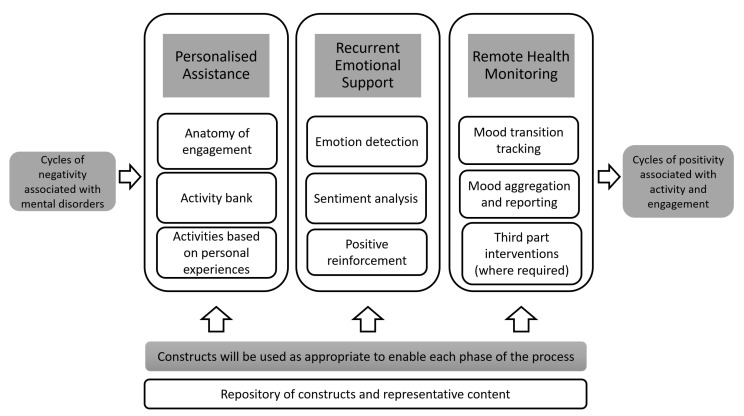
Conceptual framework of behavioural activation in an AI based chatbot.

Figure 1 illustrates the complete conceptual framework of the BA-based AI chatbot. It visualises the diverse interactions between the primary constructs identified, validated, and consolidated across the three phases (see Table 1 for a description of each construct). The flow of conversation in this framework does not attempt to replace existing healthcare services, but aims to be a companion that provides conversational support. While a counsellor might ask a patient to perform these tasks manually, the chatbot is a technological automation that simplifies the BA tasks into an efficient and scalable process. The effectiveness of the framework can be attributed to multiple factors, firstly the continuous personalised interaction with an end-user that is modelled on elements of a session carried out with a mental health professional and, secondly, each interaction composed of multiple sub-flows, where each sub-flow is drawn from the constructs. The sub-flow interlinks a logical set of constructs together to enable the segment of a conversation, for example a greeting section, a discussion about an activity, or closing the session. The sub-flows and constructs are also extensible so that the chatbot’s conversational capabilities can be adapted or extended to suit a specific domain. Each interaction provides the opportunity to schedule activities and evaluate and track mood transitions, which then leads into a cycle of planning, execution, and assessing the activities that the user is participating in and then gamifying the positive transition of the user through the mood spectrum to encourage his/her to continue being active. It should also be noted that the chatbot can detect the likelihood of critical danger or self-harm during the conversation. This detection is performed by the NLU engine in the chatbot during the conversation, by computing an alert score based on the frequency and intensity of words, phrases, and emotions representative of critical danger and self-harm [58,59]. When this score exceeds a threshold, then the chatbot immediately alerts the user that he/she is talking to a chatbot, not a human, and prompts him/her to seek urgent assistance from human experts in a formal healthcare setting. This additional layer of “help” reflects a deliberate and purposeful ethical dimension to the user interface, signalling the design has a built-in ethical construct.

The composition of a typical interaction is given below:

1. Feelings check;
2. Recap previous session/review homework
3. Agree on an agenda;
4. Check for any unexpected/unplanned activities or incidents;
5. Get out clause;
6. Do the work;
7. Summarise what was discussed;
8. Set homework;
9. Schedule next meeting;
10. Feelings check.

## 4. Design and Development of the BA-Based AI Chatbot

The conceptual framework was designed as a series of chatbot capabilities that can be grouped into three main categories, (1) personalised conversation (2) emotional support and (3) remote mental health monitoring, which are delineated in the following subsections. The user experience of engaging with the BA-based AI chatbot was a further design consideration that is articulated at the end of this section.

### 4.1. Personalised Conversation

**Figure 2:**
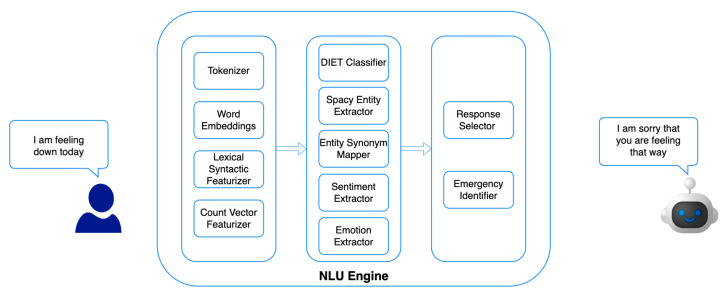
NLP engine for personalised conversations with the BA-based AI chatbot.

The personalised conversation engine (Figure 2) is based on the Rasa chatbot framework [60] with BA-oriented Natural Language Processing (NLP) modules. The NLP engine is comprised of three main components: feature extractor, intent and entity extraction, and response selection. The feature extractor is responsible for preprocessing and representation learning of the user-generated text. Intent and entity extraction is used to identify the intention of user utterances and annotate with appropriate entities. Finally, based on the intent and entities present in user utterances, a response is selected using the response selector.

In the feature extractor, multiple sparse and dense features are extracted using a lexical syntactic featuriser and count vector featuriser. The lexical syntactic featuriser creates lexical and syntactic features for a user message to support entity extraction, while the count vectoriser creates bag-of-words representations of user messages, intents, and responses. Finally, a word embedding is extracted as features using the BERT Language model [61].

Intent classification is based on the DIET classifier [62], which is a transformer-based model for simultaneous intent classification and entity extraction. A secondary entity extractor based on the SpaCy library [63] is used to extract any missing entities. An entity synonym mapper is used to map any duplicate entities identified. A DeepMoji-based [64] emotion extraction model is used for emotion extraction, and the Flair framework [65] is used for sentiment analysis.

For the final component of response selection, we developed a neural network model that identifies a predefined set of responses based on user input, intent, and entities detected in the previous component. The developed neural network model embeds user input and predefined responses into the same embedding space. The user input is concatenated with extracted entities and intents before being embedded into the embedding vector space. The model takes a similar approach to [66] with a focus on selecting the most appropriate response to the user input. A k-Nearest-Neighbour (kNN) approach was used to retrieve candidate responses. This component also contains an emergency situation detector that overrides the response selector in case the end-user responses are indicative of self-harm or critical danger.

### 4.2. Emotional Support

**Figure 3:**
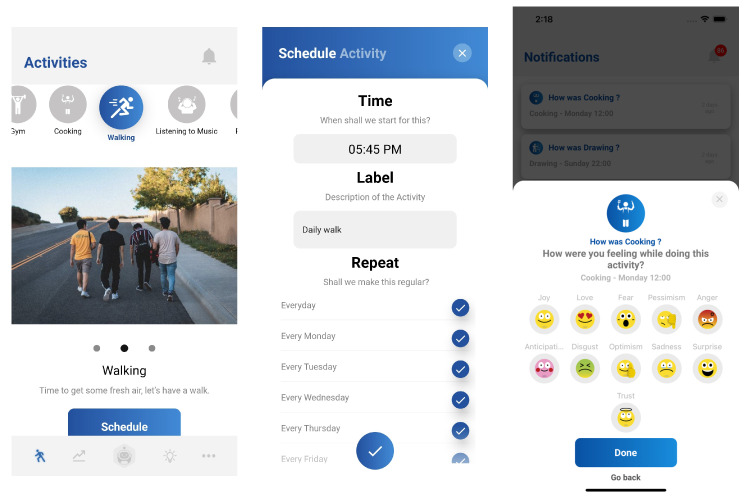
Personalised activity scheduling feature of the chatbot.

Activity scheduling is one of the core concepts of behavioural activation, which helps the user escape the vicious cycle of inactivity. Activity, whether it be sedentary or physical, has many benefits for both mental and emotional health. Research has shown the benefits can include a reduction in stress, better sleep, increased happiness, better self-confidence, increased cognitive functions, alleviated anxiety, increased energy levels, and the ability to develop and strengthen interpersonal relationships [67]. Behavioural activation focuses on changing a person’s behaviour rather than his/her thinking. The focus of BA is on getting the person to schedule activities that are meaningful to him/her. The AI conversational agent provides a feature to schedule activities from a bank of common activities. The User Interface (UI) (Figure 3) is designed to influence the user to schedule the activity in a recurring manner. The chatbot provides timely reminders to the user to follow the scheduled activity. An activity satisfaction survey is sent to the user in the form of a notification after the scheduled activity is completed. Based on the feedback received, what activities were associated with the users’ highest mood and what activities were associated with the users’ lowest mood are identified. The chatbot uses this information to recommend activities that positively impact the user’s mood. The activity scheduling, reminders, and satisfaction surveys all form part of an overall health, wellbeing, and emotional support mechanism.

**Figure 4:**
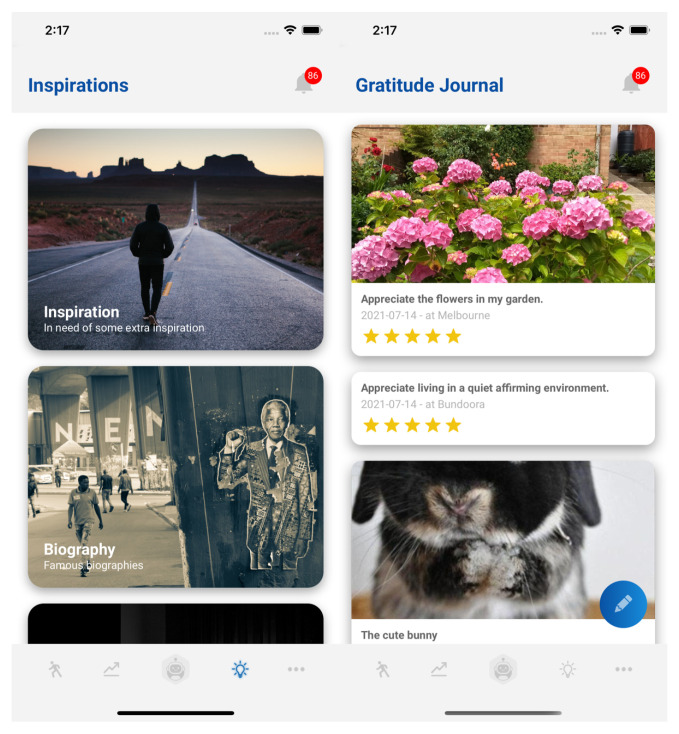
Recurrent emotional support provided by the gratitude journal and motivational content feature of the chatbot.

An individual’s moral code, values, and ethics represent what he/she finds meaningful in life and how he/she wishes to live and contribute to society. BA is able to channel these values as it encourages an individual to actively think and write down what he/she cares about and considers important in life. Being in touch with one’s values leans towards positive influences and happiness. The gratitude journal depicted in Figure 4 can be used to reflect on such thoughts and associated values, prompting recurrent emotional support based on one’s own values. This feature enables the user to record and review memorable and positive events, experiences, persons, or things in his/her life. The benefits of gratitude practice include increased happiness and positive mood, more satisfaction in life, less materialism, less likelihood to experience burnout, better physical health, better sleep, less fatigue, greater resiliency, strengthening of relationships, and encouraging the development of patience, humility, and wisdom. Emmons and Mishra [68] explored many of the above benefits and concluded that there is “considerable evidence that gratitude builds emotional resilience, social resources by strengthening relationships and promoting pro social actions”. In addition, the journal bears the ability to rate the journal entry with a star rating from one to five and attach multimedia representations of those memories, further highlighting the design’s intentional emotional support.

### 4.3. Remote Health Monitoring

**Figure 5:**
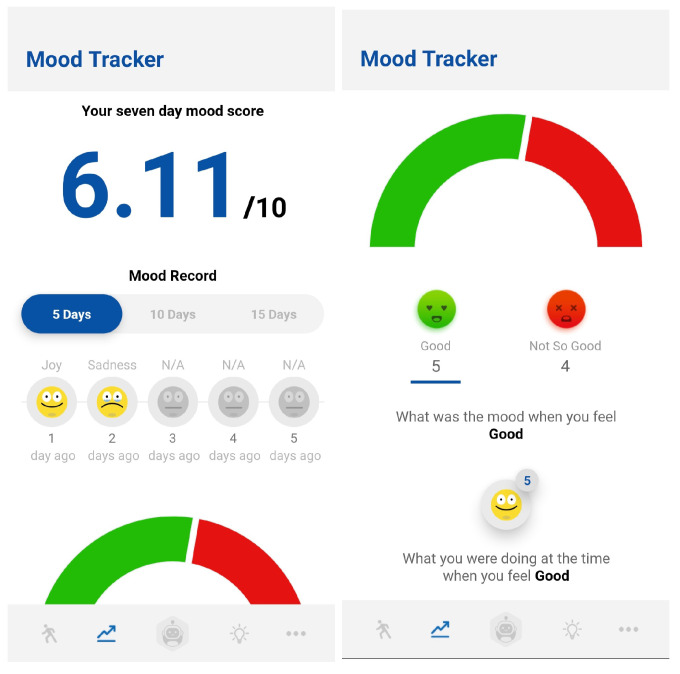
Temporal mood score and mood calendar enabling remote mental health monitoring.

Remote health monitoring is grounded in Ecological Momentary Assessment (EMA), which aims to address the limitations of the current practice of global retrospective self-reporting at predefined clinic or hospital visits. Reporting conducted during such visits is impacted by recall bias and does not detect behaviour changes over time and across contexts [69]. EMA prescribes repeated sampling of current behaviours and experiences in real time, within a natural environment. This reduces recall bias, increases ecological validity, and enables the discovery of micro-entities and micro-processes that influence behaviours in real-world settings. The manifestation of EMA as remote health monitoring in the BA-based AI chatbot is based on the emotions expressed during the check-in process and prior/post activity scheduling and undertaking. The BA-based AI chatbot is composed of state-of-the-art emotion and sentiment detection algorithms as described in the NLP engine. Bunji follows a 10-step process each time a user interacts with the app. A feelings check is conducted as the first step. During this feelings check, the user is asked “How are you feeling now?” or a paraphrasing thereof. Using pretrained emotion recognition and sentiment analysis models, a mood score between 0 and 10 is calculated (Figure 5). These mood scores are timestamped and saved to calculate a seven-day rolling average mood score. This seven-day rolling average mood score will provide the user a quantifiable metric of his/her mood in the short-term. The user also receives a Patient Health Questionnaire 2 (PHQ 2). If the PHQ2 score is above six, the PHQ2 is extended to Patient Health Questionnaire 9 (PHQ 9). These PHQ2/PHQ9 scores are also recorded. The PHQ2 score is used as an initial screening for depression symptoms. The PHQ2/PHQ9 questionnaires need to be performed with a two-week gap, but the mood score can be recorded each time the user participates in an activity or uses the app.

(1) $$7-\mathrm{day}\mathrm{average}\mathrm{rolling}\mathrm{mood}\mathrm{score}=\frac{\sum \mathrm{mood}\mathrm{scores}\mathrm{for}\mathrm{the}\mathrm{last}7\mathrm{days}}{\mathrm{number}\mathrm{of}\mathrm{mood}\mathrm{scores}\mathrm{for}\mathrm{last}7\mathrm{days}}$$

The mood calendar feature aggregates and presents the mood trend over the last 5, 10, 15, and 30 days. This allows the user to analyse his/her mood patterns and the predominant emotion for a given day. Based on this analysis, a user can identify his/her mood trend in both the short- and long-term. A further breakdown is presented to analyse what the user was doing when a particular emotion was experienced. This information can be used to identify activities that affect mood and emotion, enabling mental health monitoring across diverse periods of time.

### 4.4. User Experience Design

#### 4.4.1. Colour Scheme

Several studies report a correlation between colour and psychological functioning [70,71,72]. Specifically, Elliot [71] stated that the colour blue was reported to produce greater mental alertness. Similarly, Kurt and Osueke [70] studied the effect of colours on the moods of college students, found that blue is the favourite colour of the majority of students, and stated that blue encourages intellectual activity, reason, and logical thought. Therefore, the colour scheme of the chatbot app was chosen to be blue and white based on these studies to promote more mental awareness and positivity.

#### 4.4.2. User Interface

The chatbot user interface and user experience (UI and UX) were designed professionally to address the limitations of existing applications outlined by Alqahtani and Orji [44], Melcher et al. [45]. The use of an aesthetically pleasing user interface design and the use of matching custom-designed icons and emoticons can be noted. The chatbot logo was designed to promote approachability and trustworthiness.

#### 4.4.3. Privacy and Security

The chatbot uses encryption on the backend, and the all the data are anonymised for storage using a hash function of the username. The use of email is optional, and all the personal information such as email and name is stored in a separate Firebase (https://firebase.google.com/, (accessed on 1 January 2022)) database with no connection to the main chatbot database. All the conversation data are encrypted when storing, while the user is anonymised using a hash. Any personal information provided to the chatbot is de-identified using generic keywords. e.g., names are replaced by the keyword NAME, phone numbers by NUMBER, etc. Personal information is not used in any of the subsequent conversations or recommendations.

### 4.5. Technical Development

**Figure 6:**
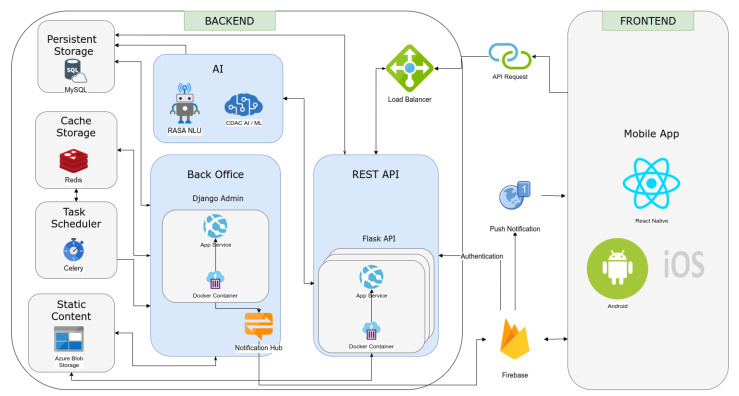
Technical architecture of the BA-based AI chatbot, which is implemented as a smartphone application on both Android and iOS operating systems.

This BA-based AI chatbot was implemented as a cross-platform smartphone application using the Python programming language [73] in the backend and the Javascript programming language [74] in the frontend. The technical architecture is depicted in Figure 6. The smartphone application was developed using the react native library (https://reactnative.dev/, (accessed on 1 January 2022)) and complied into both Android and iOS operating systems. The backend application consists of three main components, REST Programming Interface (API), back office, and AI engine. The REST API is responsible for communication between the mobile application, and it was implemented using the Flask framework [75]. The back office component is used as a Content Management System (CMS) to update and maintain static content such as inspirational quotes, biographies, and memes. The AI engine powers the conversational engine, and it hosts the NLU engine and developed AI/ML models. The Firebase service is used for user management, authentication, and push notifications. Task scheduling was performed using Celery library, and Redis is used as the cache storage. MSSQL is used as the persistent database storage. The complete chatbot implementation is hosted on the Azure cloud service as a microservice using Docker containers [76]. Several cloud computing services are to host the Bunji backend such as Azure App Services and Azure Blob Storage.

## 5. Participatory Evaluation

The participatory evaluation of the completed chatbot is reported in this section. The pilot study was conducted in Australia; therefore, we named the chatbot Bunji, which means “a close friend” in the Indigenous Warlpiri and other languages of the Northern Territory and northern Queensland [77]. Bunji is available on the Google Play Store (https://play.google.com/store/apps/details?id=au.edu.latrobe.cdac.bunji, (accessed on 1 January 2022)) and Apple App Store (https://apps.apple.com/us/app/bunji-a-good-friend-to-chat/id1524154633, (accessed on 1 January 2022)). The pilot study period was set to eight weeks, during which time it was downloaded and used by 318 individuals across the world, which was a surprising result considering the pilot was only “meant” for Australian users.

The pilot study was carried out using anonymised text and mood-related data generated by these users during the period of 1 June 2021 to 1 August 2021. The data consisted of activity scheduling details, activity feedback mood scores, PHQ9 scores, and conversational data. An online questionnaire was designed and issued to selected users following this eight-week period of use. The users were selected based on the following inclusion criteria: (1) use a smartphone with either Android or iOS operating system, (2) install Bunji app from either Google Play Store or Apple App Store, (3) agree to Bunji’s terms of service and privacy policy, (4) use the app between 1 June 2021 and 1 August 2021, (5) use the app to answer at least two valid PHQ2 surveys, and (6) use the app to perform at least two “feelings check-ins”. The last two inclusion criteria (i.e., completed a minimum of two feelings checks and two PHQ surveys within the study period of eight weeks) signify a potential user who is likely to benefit from the BA and AI features of Bunji. Users must voluntarily submit their feelings checks and PHQ2 surveys while using the app. This inclusion criteria yielded 34 eligible participants. We obtained their consent for two experimental studies that evaluated the functionality and use of the app. The third study also included participants who installed the app, but did not use it a single time. The results of all three studies were fully anonymised, and we only report aggregated insights. The three studies were named as follows: (1) Study 1—formulating a mood improvement metric to evaluate the effectiveness of Bunji’s recurrent emotional support, (2) Study 2—impact analysis on the effectiveness of personalised conversation, and (3) Study 3—focus groups to evaluate the effectiveness of remote mental health monitoring and overall feedback on Bunji.

### 5.1. Study 1—Mood Improvement

This study was composed of a one-group pretest–posttest quasi-experimental design to measure the effectiveness of Bunji’s emotional support features. We formulated a metric of mood improvement that can be attributed to Bunji’s recurrent emotional support. The metric is based on two data points of the feelings checks: initial feeling check (“pre-usage”) and the last feelings check (“post-usage”), and the two data points have to be at least seven days apart. Next, we used the Shapiro–Wilk test to evaluate and confirm that the data were not normally distributed. The *p*-value obtained from the Shapiro–Wilk test was significant for the pre-usage sample ($p=3.13\times 10^{-5}$) and post-usage sample ($p=1.50\times 10^{-5}$). Therefore, we concluded that the data were not normally distributed and that the Mann–Whitney U test should be used to analyse the two samples. We performed a one-sided Mann–Whitney U test to test whether the mood score improved from pre-usage to post-usage. Table 2 contains the summary statistics of the tests. As the *p*-value obtained from the Mann–Whitney U test was significant (U=434.5, p=0.03), we concluded that the mood score significantly improved from pre-usage to post-usage.

### 5.2. Study 2—Impact Analysis of Personalised Conversation

From the selected sample, 88.26% (30/34) of users scheduled an activity and followed the schedule during the study period. On average, users scheduled three activities per week. On average, a user engaged in a conversation with Bunji seven times during the study period. In total, 44.12% (15/34) used the gratitude journal to keep a note of a positive event, experience, person, or thing in their lives. There were 85.29% (29/34) of the users who used the inspiration section, while 29.41% (10/34) of the users saved one or more inspirational posts.

**Figure 7:**
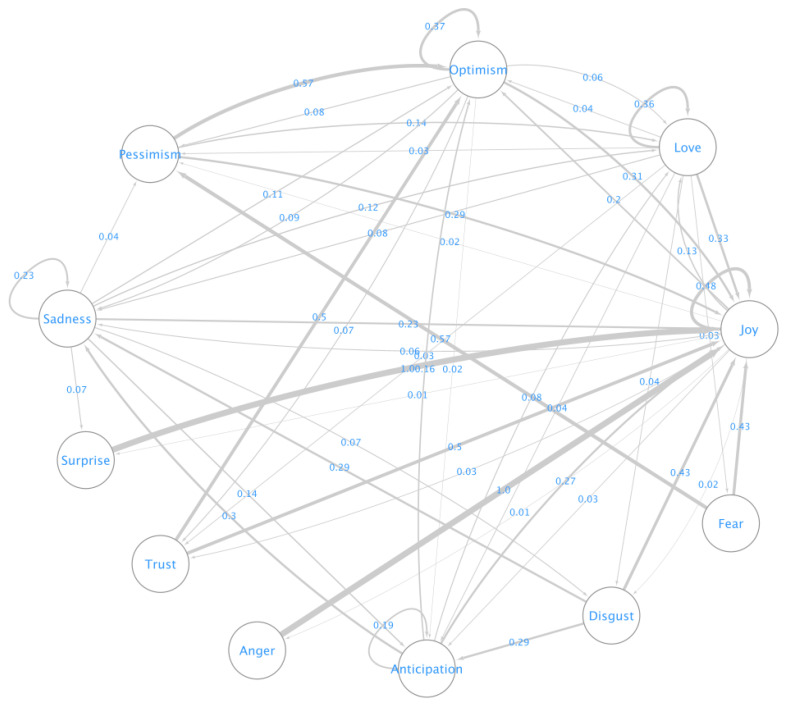
Emotion transition graph of users during the study period.

Bunji uses 11 emotions to detect mood, and they are anger, anticipation, disgust, fear, joy, love, optimism, pessimism, sadness, surprise, and trust. During each interaction, Bunji makes note of the users’ emotion and derives its transition. Figure 7 depicts the mood transitions of the users during the study period. Using in-degree centrality and the pagerank algorithm in the mood transition graph, we can identify that the joy emotion had the most in-degree centrality of 1.1 and the highest page rank, while fear and anger had the least in-degree centrality of 0.1 and the lowest page rank. This measure identifies that most users end up being happy regardless of the initial emotion they present.

**Figure 8:**
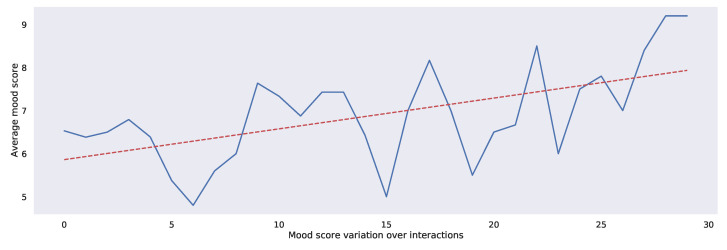
Mean mood score of all study participants over the first nine interactions with Bunji (An interaction is when a user initiates a chatbot function. This can be a conversation, activity, or other chatbot feature). The blue line graph connects the average mood score across each interaction, while the red dotted line depicts the ordinary least squares regression line for these points.

**Figure 9:**
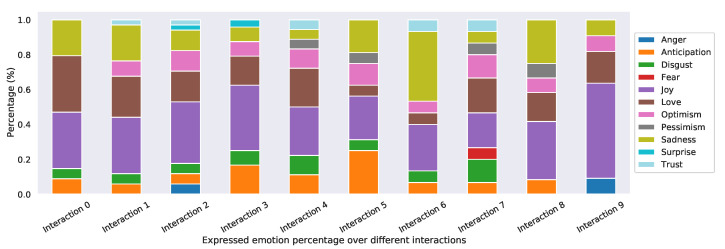
Emotion expressed as a proportion over different interactions (An interaction is when a user initiates a chatbot function. This can be a conversation, activity, or other chatbot feature.).

Similarly, Figure 8 presents the average mood score for the *i*th interaction with the chatbot. Users display a recurrent mood score pattern of highs followed by lows during the various interactions. However, the lows become higher, and the highs become even higher as the number of interactions increased, i.e., the mood score was trending upwards in the long run. Figure 9 breaks down the percentages of emotions expressed during the first 10 interactions with the chatbot. Joy, love, and optimism accounted for most interactions in which the average mood score was high. Similarly, sadness, disgust, and anticipation accounted for most interactions in which the average mood score was low.

### 5.3. Study 3—Qualitative Feedback on Remote Mental Health Monitoring

In this study, a feedback questionnaire containing 10 questions was presented to all individuals who installed the application. This included those who did not use the application at all because we wanted to understand the design/functional limitations that led to such a decision. A thematic analysis was conducted on the collected feedback. The analysis discovered the following three main groups in terms of frequency of use.

Group 1: Installed the app and used all features (51.62%).
Group 2: Installed the app and used some features (38.71%).
Group 3: Installed, but did not use it at all (9.67%).

The findings of Group 3 surrounded the risks of privacy breaches and also being time poor. Interestingly, respondents commented that the prompts and reminders built into the app to schedule activities do make a difference to help users feel better and that it refocuses attention towards self-care. This group also made comments pertaining to the onus on the individual to hold themselves accountable, which is a difficult adjustment to make. All users found the interface easy to use and that the gratitude journal was effective in terms of portability and availability. The responses related to Group 1 reported extremely positive comments that were indicative of how Bunji was used for remote health monitoring and support. Some of these responses were:

1. “By using the app, I am more aware of how my moods fluctuate. It also made me think about what I am grateful for, which alleviated some negativity I was experiencing at the time”
2. “Mood tracker is great to be able to give to my medical practitioner—If my psychologist or doctor asked me to use this as a monitoring tool in between sessions, I would be more likely to engage”
3. “Bunji lets me feel courageous”

## 6. Conclusions

As conversational agents are becoming a readily available platform for many service providers, the benefits in the healthcare domain are emerging. The design and development of our BA-based AI chatbot, followed by its participatory evaluation, confirmed its effectiveness in providing support for individuals with mental health issues. With an ever-increasing demand for healthcare service providers and mental health issues being at the forefront of healthcare challenges, our “Bunji” has the potential to be scaled up and rolled out in the healthcare domain to support front-line workers and the community as a whole. Prior to such a deployment, we need to conduct and report on long-term evaluation of functionality and the impact of regular usage of the chatbot across diverse cohorts representative of the socio-demographic interest groups. This is a primary limitation of this study that we intend to address as part of our future work. We also plan the following innovations as future work: creation of a community of Bunji users with like-minded interests who can engage in group activities, Bunji being able to find “Chat Pals” in this community, which is ideal for people who are more introverted and live by themselves, and extending the gamification of Bunji for visually setting and tracking goals against a community benchmark and one’s own track record. We are also working on expanding the activity banks to include activities, inspirations, quotations, and workshops, as well as enhancements to the language model for more fun/inspirational human-like conversations with a range of responses that suit diverse demographics (e.g., emojis and memes). We will also consolidate the code of ethics with opt-in options for users to provide more information, which will improve the personalised conversations. In conclusion, mental healthcare and treatment are major challenges inhibiting the health and well-being of contemporary human society, and this study contributes the technological innovation of an intelligent chatbot with cognitive skills for personalised behavioural activation and remote health monitoring.

## Figures and Tables

**Table 1:** The primary constructs of the BA-based AI chatbot.

Primary Construct	Description
Anatomy of engagement	Derives structure from typical conversation that a mental health practitioner would have with patients.
Emotion detection and sentiment analysis	Discern an individual’s emotional disposition from his/her speech, based on either three emotions (positive, negative, and neutral) or eight emotions (anger, fear, sadness, disgust, surprise, anticipation, trust, and joy).
Mood transition tracking	Evaluate and monitor the user’s mood through the use of specialist tools such as PHQ2 and PHQ9. Derive an evidence-based understanding of the transition of a user through moods.
Mood aggregation and reporting	Summarise and synthesise mood scores and all emotion expressions with intensity scores across multiple granularities, daily, weekly, monthly, and yearly.
Activity bank	Provide a bank of common activities that can be used to personalise a user’s experience toward becoming active. Will also provide a base from which the community can be built looking into which activities will typically improve mood.
Personalised experiences	Use evaluation-based methods to understand the mood of a user following the completion of an activity, i.e., how did participating in an activity make the user feel.
Positive reinforcements	Contribute towards recurrent emotion support through inspirations drawn from a compilation of quotations, imagery, inspirational, and emotional journeys.
Third-party intervention	Be cognizant of indications of self-harm by monitoring conversational cues and direct the users to formal healthcare services and support.

**Table 2:** Results of Experiment 1 on mood improvement.

Users with at Least 2 Feelings Checks and PHQ2	Pre-Usage	Post-Usage
**Number of users (N)**	34	34
**Mean mood score**	5.79	7.38
**Median mood score**	6.50	8.00
**Shapiro–Wilk statistic (** ***p*** **-value)**	$0.80\;(3.15\times 10^{-5})$	$0.78\;(1.50\times 10^{-5})$
**Mann–Whitney U (*p*-value)**	-	434.5(0.03)
